# Obsessive compulsive disorder: Current understanding and future directions

**Published:** 2009

**Authors:** M. V. Ashok

**Affiliations:** Department of Psychiatry, St Johns Medical College, Bangalore mysoreashok2003@yahoo.co.uk.


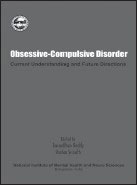


ECT and OCD are arguably two large areas of work where the prevalent clinical practices and research endeavors at the National Institute of Mental Health and Neuro Sciences, Bangalore have achieved nationwide acceptance. The recent international conference on OCD held at NIMHANS in November, 2007, was attended by a record number of delegates. It is fitting that the proceedings of this conference be compiled and made available as a key resource for clinicians and researchers. The release of this book at the time of the conference is indeed a feather in the cap for organizers of the conference and the editors.

The book has 11 chapters. Two are devoted to nosological issues. Three chapters focus on neurobiology while three other chapters focus on treatment issues and long term outcomes in adult OCD. Three chapters being devoted to Childhood onset OCD is not only a reflection of the research interests of the editors, but also signifies the growing developmental perspective in OCD research.

The paper by DJ Stein highlights two perspectives on conceptualizing OCD spectrum disorders – the classical approach and the critical alternative. The latter points out that the concept reflects researchers more than the topic! The author goes on to argue that it ‘may be possible to construct an integrative approach to classification which is based on cognitive-affective science’. At times tenuous and at times brilliantly discursive, this chapter attempts to locate OCD amongst a group of potentially related disorders.

The other article on nosology by David Mataix-Cols and JF Leckman selectively explores heterogeneity within OCD from genetic, neurobiological and developmental perspectives. It calls for exploring heritability of different symptom dimensions and associated co-morbidities. This chapter by a person leading DSM-V- views on OCD is well written and questions constitution of the core features of this disorder. This one is again for the serious reader though.

The two chapters on the neurobiology and genetics of OCD by Venkatasubramanian Ganesh and Valsamma Eapen respectively are both helpful reviews of various studies in the area and should warm the cockles of post graduate students. They both remark on the heterogeneity within OCD and the prevailing need for further exploration of symptom dimensions using modern tools of cognitive neuroscience. Collating the relatively sparse literature on immune factors in OCD, the chapter on immune factors in OCD by Sagnik Bhattacharya speculates on the role of infections and immune mechanisms leading to OCD in genetically vulnerable individuals. The three chapters in all testify to the well established emphasis on the biological factors in OCD research.

Janardhan Reddy and Suresh Badamath review both naturalistic follow up studies as well as the more recent longitudinal studies and remark on the limited data. They conclude that prognosis of OCD is ‘not as bleak as it is widely portrayed’. This chapter is an excellent resource on the topic and expands on some Indian data. The chapter on management of treatment resistant OCD by Sumant Khanna defines treatment resistance and summarizes the assessment necessary. The recommendations for clinical practice are direct, pragmatic and supported by the thought processes of a pioneering researcher and well known clinical expert in the area. Indian clinicians and researchers alike, have plenty to learn from these two chapters.

The chapter on psychotherapy in the treatment of OCD, current status and future directions by Paul Salkovskis, a well known proponent of CBT in OCD, is a delightful read – right from its reference to the first case of OCD treated by psychological methods way back in 1966 all the way to its emphasis on the need for expanding psychological services in a planned manner today. There is an excellent synopsis of CB theory underlying its use in OCD with a key focus on maintaining mechanisms. The treatment processes are delineated at length and should serve both practitioners and post graduate students well. The author's views on the need to emphasize, in a clinical context, on the ineffectiveness of reassurance seeking/giving may intrigue or even challenge Indian clinicians. While the author grandly asserts the changing scenario – from stigmatizing pessimism to heady optimism—Indian readers may lament the absence of trained personnel for delivering such services.

Childhood OCD—what is unique about it? - is a scholarly review that comes up with a list of clinical, familial and co morbid aspects that qualify OCD in childhood. It concludes that outcome in childhood OCD is better than previously recognized and generally better than that in adults. This valuable review is well supplemented by Indian data on childhood OCD by the editors of the book. From their own pioneering data, they remark on the favorable course and absence of familiality as well as PANDAS in our context. The chapter reviewing CBT in childhood OCD by the team at NIMHANS lists the scanty literature in the worldwide literature and poses questions. This chapter's utility is limited by the virtual absence of Indian data.

The book is well referenced and will remain a valuable resource base for some years to come and certainly abets the editors' desire to ‘boost research in this area'. The book signifies the maturing of OCD services and research at NIMHANS and the editors’ willingness to confidently position themselves in the international community of OCD researchers. One hopes that the success of the conference and the publication of this resource book would propel the team towards further critical exploration of the OCD construct in an arguably ritualized Indian society.

The preface refers to the book erroneously as a monograph, which by definition should be an extended essay by a single author or a defined group of persons like in a department etc. The striking lacuna is the absence of a unifying chapter by one or both editors that could have summarized the contents of the book for the reader in a hurry. Also, Figures and diagrams may have helped the cause of engaging busy practitioners' attention. That said, this octavo sized book with 292 pages makes for easy carry and read. Its worth would have been enhanced by listing the titles/authors of various papers presented in the poster sessions as well.

